# Age-associated changes of the intrinsic nervous system in relation with interstitial cells in the pre-weaning goat rumen

**DOI:** 10.18632/aging.102076

**Published:** 2019-07-14

**Authors:** Yu Liang, Imran Tarique, Waseem Ail Vistro, Yifei Liu, Ziyu Wang, Abdul haseeb, Noor Samad Gandahi, Adeela Iqbal, Siyi Wang, Tianci An, Huan Yang, Qiusheng Chen, Ping Yang

**Affiliations:** 1 MOE Joint International Research Laboratory of Animal Health and Food Safety, College of Veterinary Medicine, Nanjing Agricultural University, Nanjing, Jiangsu 210095, China; 2 College of Animal Science and Technology, Nanjing Agricultural University, Nanjing, Jiangsu 210095, China

**Keywords:** intrinsic nerve, interstitial cells, rumen, goat

## Abstract

In this study, we investigated the neural changes and their relationships with interstitial cells (ICs) in the rumen of pre-weaning goats by transmission electron microscopy, western blot and immunofluorescence (antibody: general neuronal marker-Protein Gene Product (PGP9.5)/ IC marker-vimentin). The immunofluorescence results showed that PGP9.5-positive reaction was widely distributed in neuronal soma (NS) and nerve fibre (NF). The NSs were observed in the ganglia of the myenteric plexus (MP) but not in the submucosal plexus. The mean optical density (MOD) of the whole of PGP9.5-positive nerves and the protein expression level of PGP.5 in the rumen wall both decreased significantly with age. However an obvious increase MOD of PGP.5-positive NFs within the rumen epithelium were observed. In the MP, the nerves and ICs were interwoven to form two complex networks that gradually tightened with age. Furthermore, NSs and nerve trunks were surrounded by a ring-boundary layer consisting of several ICs that became physically closer with aging. Moreover, ICs were located nearby NFs within the ML, forming connections between ICs, smooth muscle cells and axons. This study describes the pattern of neural distribution and its association with ICs in the developing rumen which shed light on the postpartum development of ruminants.

## INTRODUCTION

The enteric nervous system (ENS), known as intrinsic nervous system (INS) comprises the intrinsic neural circuits of the gastrointestinal tract (GI). It regulates most aspects of GI physiology such as peristalsis, blood supply to the gut wall and secretion [1]. The complex architecture and function of the nervous system arises from neurogenesis and development [2, 3]. Intrinsic neural networks are derived from neural crest cells that colonize the gut during embryogenesis and give rise to enteric neurons and glial cells [4]. Meanwhile, mesoderm mesenchymal cells also differentiate into smooth muscle cells and interstitial cells, which is important for the postpartum development of INS for their cross-talk between smooth muscle cells/interstitial cells and INS frequently [5, 6]. However, the enteric neurogenesis lasts for several weeks after the birth [7, 8].

**Figure 1:**
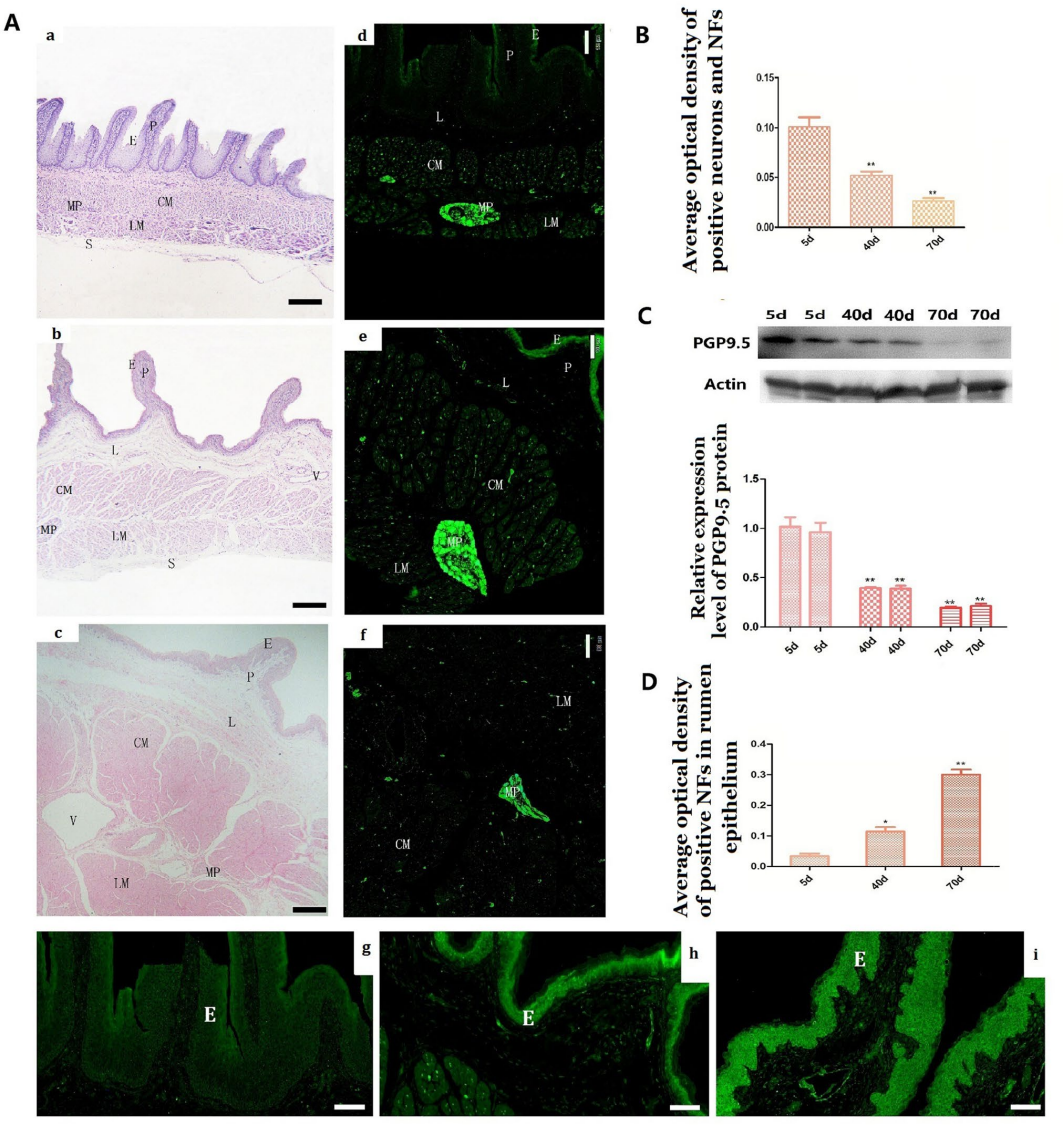
Histologic changes and the expression of PGP9.5 immuno-positive and its protein expression in the rumen wall of goat during pre-weaning (**A a–i**). Histologic changes of rumen wall with age (**A–a**, 5 days group; **A–b**, 40 days group; **A–c**, 70 days group). The distribution of PGP9.5-immunoreactivity in the rumen wall (**A d–f**) and MOD of PGP9.5-positive NFs in the mucosa (especially in the epithelium) with age (**A–g**, 5-day group; **A–h**, 40-day group; **A–i**,70-day group). The reverse changes of PGP9.5 positive NFs in the rumen wall (**B**) and in the rumen papilla (**D**) from new born to weaning. Western blot analysis of PGP9.5 in protein extracts of rumen segments from pre-weaning goats (N=4 per group) (**C**). β-actin was used as a loading control. Protein signals were quantified using densitometry analysis. Data are reported as mean ± SEM. Statistical significance was determined by one-way ANOVA followed by Newman-Keuls post-hoc test for multiple comparison (panel **A**) *P<0.05 vs 5d; **P<0.01 vs 5d. CM, the circular muscle layer; E, epithelium; P, papilla; L: lamina propria; LM, the longitudinal muscle layer; MP, the myenteric nerve plexus; CM, circular muscle layer; V, vessel. (Bar =160 μm in **A a–c**; Bar =100μm in **A d–f**; Bar =35μm in **A g–i**.)

The rumen, a specialized digestive organ of ruminants, plays a key role in the absorption and digestion of nutrients. Most of the important physical and biochemical processes occurring during rumination take place in the rumen [9]. Noteworthy, the organs have been formed *in utero.* However, to ensure appropriate regulation of the distinct functions, the organs have to continue to develop and to differentiate to adapt to environmental changes after the birth of the animal [10–12]. The embryonic stomach of goats, like other monogastric animals, originally developed from a spindle swelling of the primitive foregut. The rumen of breast-fed newborn lambs has little digestive capacity, and it develops rapidly only after grazing. With the increase of age, the intake of solid fiber feed increases gradually. The volume and the proportion of the rumen also increase rapidly, and the function starts to improve. Therefore, general scholars combined feeding types with the characteristics of digestion and the structure of the goat rumen, dividing its postembryonic development into three typical stages [9, 13]: the non-ruminating stage feeding with milk or a milk substitute that enter the abomasum directly avoiding the degradation and destruction of rumen microorganisms (newborn to 3 weeks), and in which the rumen volume slowly expands; the transitional stage feeding with liquid feed and starter combination (3 to 8 weeks), and in which the rumen volume expands rapidly; and the ruminating stage feeding with fibrous that were digested mainly through rumen microorganism fermentation (after 8 weeks), and in which the rumen volume is stable. Moreover, it has been demonstrated that the optimal weaning period for the rumen is 2–3 months. From birth to weaning, the rumen has undergone tremendous changes in these three stages. The development of the rumen is mainly related to histological changes, such as the increase of the thickness of the rumen wall and the enlargement of the rumen papilla (Figure 1A-a,b,c).

It is well known that IC is essential for the INS by supporting and feeding neurons, regulating synaptic transmissions and mediating communication between the nervous and immune systems [14–24]. Studies on ruminant INS are sparse although it is an important component of the GI. Therefore, in this study, we focused on the postpartum development of the goat rumen INS and its relationship with IC during the pre-weaning period. Our experimental setup was composed of three groups of pre-weaning goats: 5 days (liquid feed stage), 40 days (transition stage), and 70 days (fiber feed stage).

## RESULTS

### Age-related histologic changes of the rumen and the nerve distribution from birth to weaning

**Figure 2:**
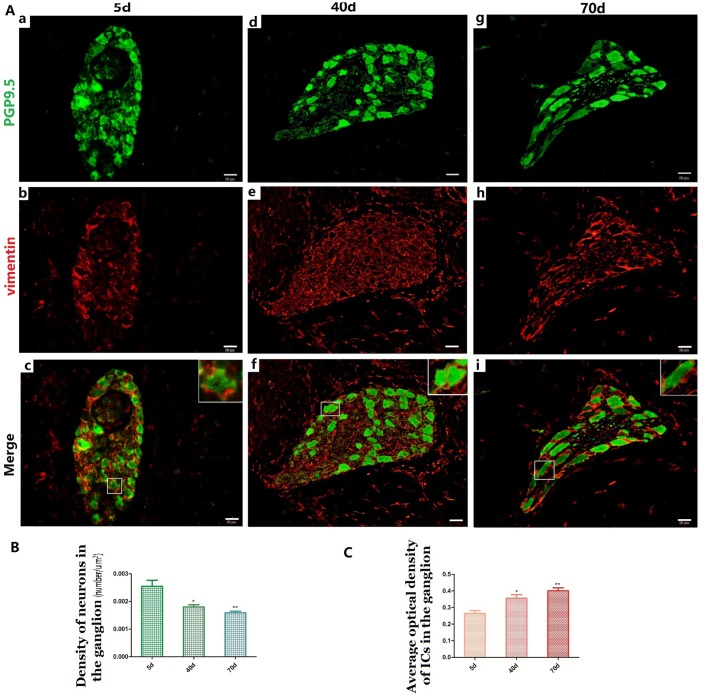
Relationship between PGP9.5-positive neurons (green) and ICs (red) in the ganglia of rumen MP (**A a–i**) with age. The neural and IC networks were observed and interweaved with each other in the rumen wall. Higher magnification views of the square frames in (**A–c,f,g)**, the somata and protuberances of two or three ICs, close to each other, formed a ring-boundary layer surrounding each NSs in the ganglia. A decreased density was observed in NS of the ganglia (**B**), while the MOD of ICs significantly increased in the ganglia (**C**). (Bar = 20 μm **A a–i**.)

**Figure 3:**
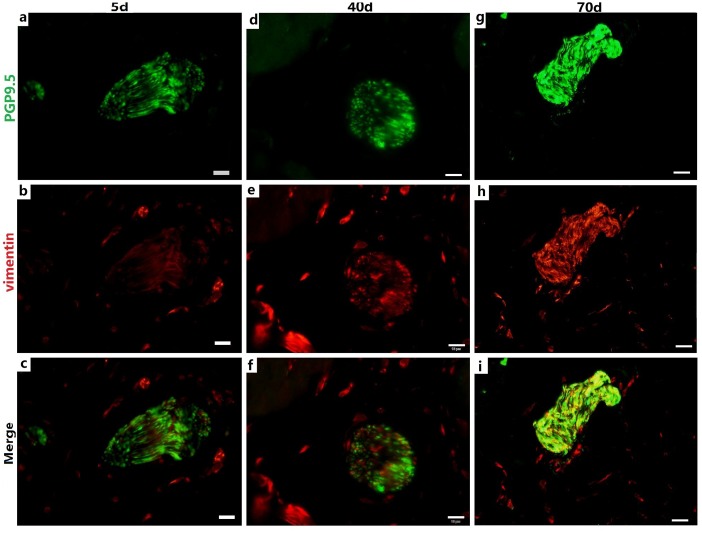
**Relationship between PGP9.5-positive nerve trunks (green) and ICs (red) in the rumen MP with age.** Several ICs surrounding a nerve trunk formed a ring-boundary layer, while most ICs interlaced with PGP9.5-positive NFs in the internal nerve trunks. The ICs ring-boundary layer tends to be seamless and gradually closer to the nerve trunk. (Bar =10 μm **a–i**.)

In the 5-day group, PGP9.5-immunoreactivity was mainly observed in NFs within mucosa (epithelium, lamina propria), muscular layer (CM and LM) and MP of the rumen, while almost all PGP9.5-positive ganglial NSs appeared as small groups of cells connected by their projections in the MP (Figure 1A-d). PGP9.5-positive NSs were irregular or polygonal in shape with clearly visible projections (Figure 2A-a). Interestingly, no PGP9.5-positive NSs were found in the submucosal plexus (Figure 1A-d). Some fairly positive thick NFs (nerve trunks) were found in the MP (Figure 3A-a) and meandered showing branch-like structure through CM and LM (Figure 5A-a; 6A-a). In the two smooth muscle layers, few nerve trunks and numerous varicose terminals were associated with the smooth muscle bundles (Figure 5A-a, 6A-a). In the lamina propria, only a few pockets of PGP9.5-positive NFs were observed. These were mainly located in the vicinity of blood vessels, while they were almost absent in the epithelium (Figure 1A-g).

**Figure 4:**
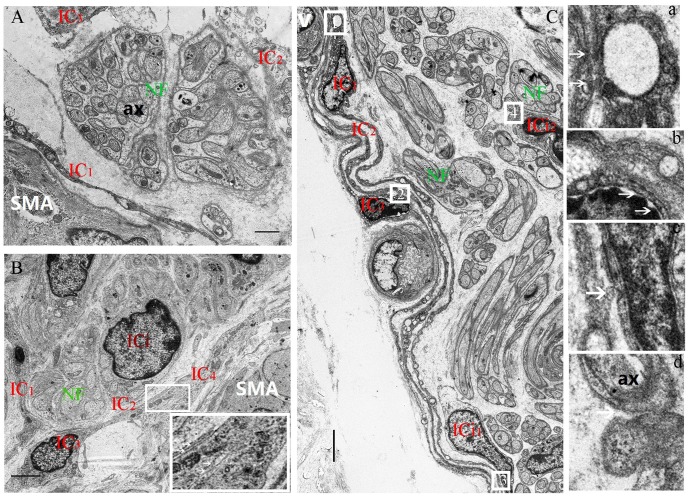
**Decrease of the space between the nerve trunk and the ICs ring-boundary layer formed by several ICs in MP with age.** In the 5-day group, the loose boundary layer surrounded the nerve trunk (**A**). In the 40-day group, the boundary layer was obviously close to the nerve trunk (**B**). Higher magnification views of the square frames in (**B**), the close relationship among IC_2_-IC_3_-IC_4_. In 70-day group, the close-knit ring-boundary layer was closest to the nerve trunk. Higher magnification views of the square frame 1,2,3,4 in (**C**), the several ICs with close connections at the arrows between IC1-IC_2_(a), IC_2_-IC_3_(b), IC_3_-ICi_1_(c), and the same close connections at the arrow between ICi_2_-NF (d) was observed in the rumen MP. A vessel blood was also found close to the ring-boundary. IC, interstitial cell; ICi, interstitial cells in the nerve trunk; NF, nerve fiber; SMA, Smooth muscle; ax, axon; V, vessel blood. (Bar = 1μm in **A**, Bar = 4μm in **B**; Bar =3 μm in **C**.)

**Figure 5:**
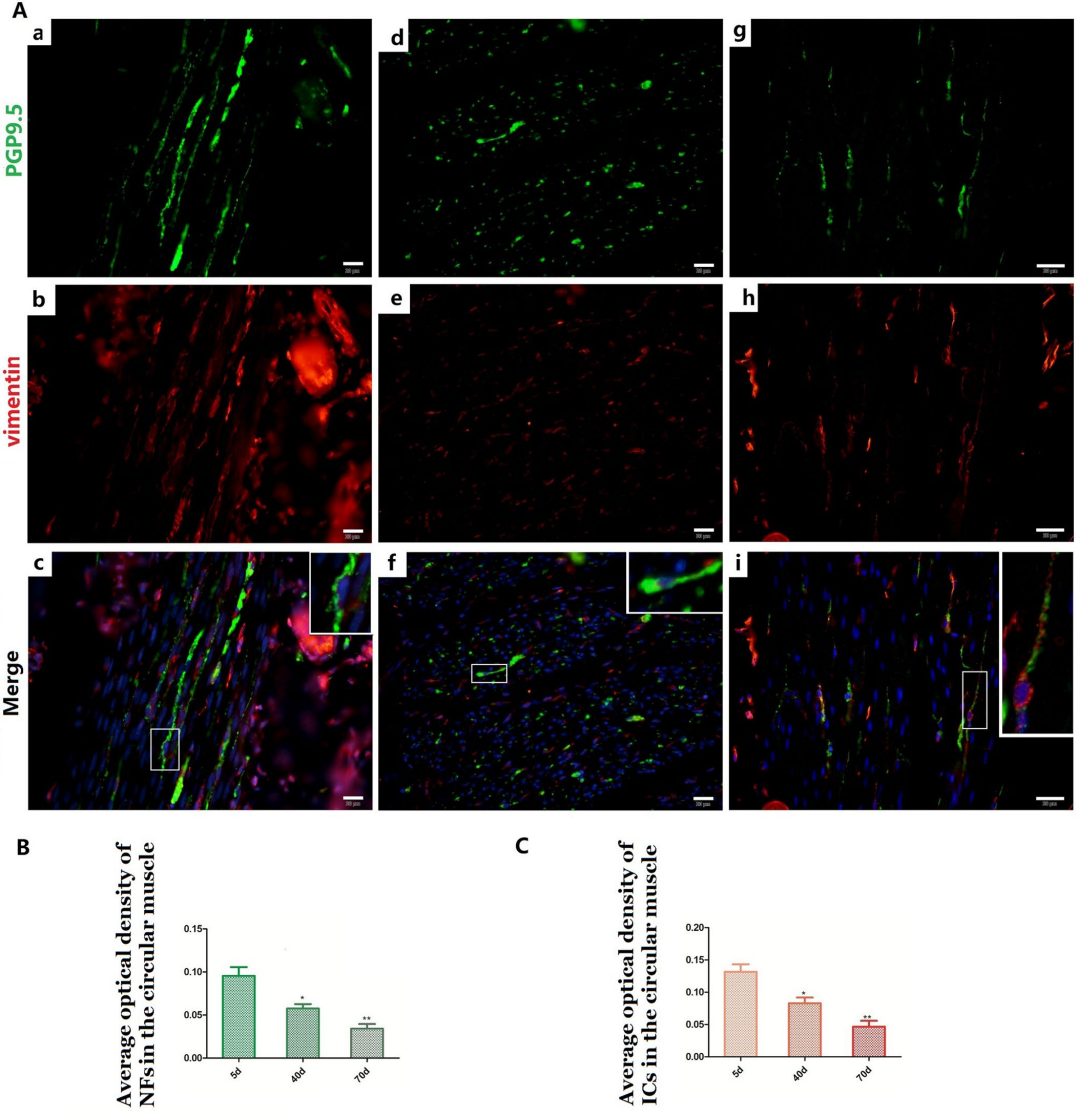
**Relationship between PGP9.5-positive NFs (green) and ICs (red) in rumen CM with age.** Higher magnification views of the square frames in (**A–c**,**f**,**g**). The cytoplasm and protuberances of the ICs were wrapped by the varicosities with PGP9.5. The MOD of PGP9.5 positive NFs (**B**) and ICs (**C**) in the CM with age, both of them decreased. (Bar = 20 μm in **A a–i**).

**Figure 6:**
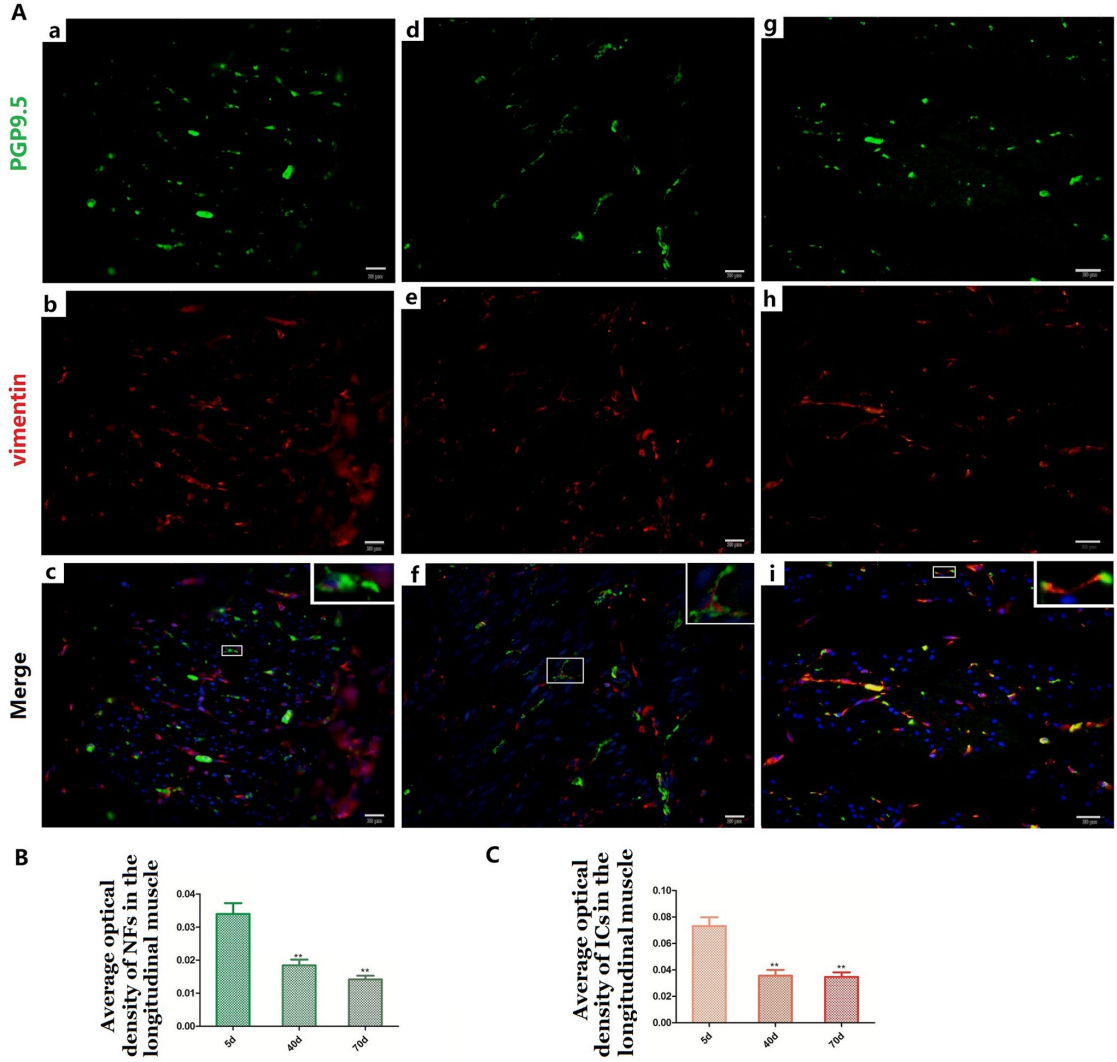
Relationship between PGP9.5-positive NFs (green) and ICs (red) in the LM in 5-day (**A a–c**), 40-day (**A d–f**) and 70-day (**A g–i**) groups, respectively. Higher magnification views of the square frames in (**A–c,f,g**). The cytoplasm and protuberances of the ICs were wrapped by the varicosities with PGP9.5. The MOD change of PGP9.5 positive NFs (**B**) and ICs (**C**) in the LM with age, both of them decreased. (Bar = 20μm in **A a–i**).

The distribution pattern of PGP9.5-positive neurons in the 40-day group was similar to that of the 5-day group (Figure 1A-e). The MOD of PGP9.5-positive NFs and the protein expression level of PGP9.5 both significantly decreased with age, along with tube wall expansion and smooth muscle cell growth (Figure 1B, 1C). Noteworthy, the amount of PGP9.5-positive NFs increased in the lamina propria at this developmental stage and these were mainly located around blood vessels and even scattered within the epithelium (Figure 1A-h, 1D). In the 70-day group, NSs were mostly distributed in MP ganglia (Figure 1A-f). PGP9.5-positive NFs were significantly increased and widely distributed in the mucosa, especially in the epithelium (Figure 1A-i, 1D). The PGP9.5-positive NFs were distributed along the blood vessels and extended to the epithelium. In all the examined preparations, the number of PGP9.5-positive NSs per ganglion was lower in the 40-day and 70-day groups compared to the 5-day group (Figure 2B).

### Relationships between NSs, NFs and ICs in the MP

The IC marker vimentin was used to identify ICs and the ICs network with extending projections developed with age (Figure 2A-b,e,h). In ganglia, the developing neural network was composed of NSs and small NFs (Figure 2A-a,d,g) and each PGP9.5-positive NS was closely surrounded by a ring-boundary layer composed of several ICs (Figure 2A-c,f,i). In the internal of nerve trunk the ring-boundary layer of ICs covering around the whole nerve trunk, while each NF were tightly interlaced with IC in the internal nerve trunk (Figure 3). Ultrastructural, the ICs surrounding nerve trunks displayed projections gradually extending and lengthening with age. These eventually formed most seamless ring-boundary layer through by the homotype connections among ICs (Figure 4A–C). NFs were closely associated with ICs all along rumen development, and this association became tighter with age (Figure 2, Figure 3, 4). In the MP, the density of PGP9.5-positive NSs in ganglia decreased (Figure 2B) whereas the MOD of vimentin-positive ICs increased significantly during development (Figure 2C).

### Relationships between NFs and ICs in the muscle layer

NFs and ICs were scattered in the muscular layer, close or parallel to each other, forming a relatively sparse distribution pattern within the pre-weaning goat rumen (Figure 5 and Figure 6). The MOD of PGP9.5-positive NFs and vimentin-positive ICs both decreased in the ML (Figure 5B, Figure 6B and Figure 5C, Figure 6C), while their projections gradually extended from 5 days to 70 days (Figure 5A-a,b,d,e,g,h and Figure 6A-a,b,d,e,g,h). This indicates that some of the NFs developed while the others were gradually dispersed. A similar pattern was also observed within both the CM and and the LM.

**Figure 7:**
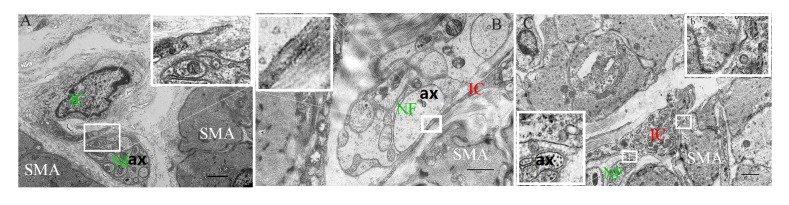
The intimate relationship between the nerve trunk and the ICs in the muscle layer of 5-day (**A**), 40-day (**B**) and 70-day (**C**) groups, respectively. Higher magnification views of the square frame in (**A**). The IC projections were close to the nerve trunk in the 5-day group. Higher magnification views of the square frame in (**B**), the close connection between the axon and the IC projection. Higher magnification views of the square frames in (**C**). The close connection not only between the axon and the IC projection (a), but between the muscle and the IC soma (b). IC, interstitial cells; NF, nerve fibers; SMA, Smooth muscle; ax, axon. (Bar = 2μm in **A**, Bar = 1μm in **B**,**C**.)

Most of the ICs (Figure 5A-b,e and Figure 6A-b,e,h) were parallel to the NFs, forming a sparse network (Figure 5A-c,f,I and Figure 6A-c,f,i) that became even more sporadic while aging since the NFs and ICs were gradually dispersed (Figure 5, Figure 6). The cytoplasm and protuberances of the ICs were wrapped by the varicosities with PGP9.5. A close connection between the axon, ICs and muscle cells was observed (Figure 7). As opposed to the pattern in the MP, the decrease of ICs was concomitant with the decrease of NFs in the ML. Hence reflects the associated relationship between NFs and ICs within the ML and MP, respectively.

## DISCUSSION

Our study, for the first time, revealed the age-related neural distribution patterns in the developing rumen of pre-weaning goat. Moreover, the relationship between NSs /NFs and ICs was also investigated.

Neural distribution pattern in the rumen wall of goat during the early pre-weaning was different from that reported in the forestomach of ruminants [25–30] or the GI of non-ruminants [8, 31–33]. Morphologically, the goat rumen presented four major layers already observed in non-ruminants intestine: the mucosa, submucosa, muscularis externa (outer LM and inner CM) and serosa [34–36]. Previous studies have observed that rumen papillae length, width and area increase with proceeding ruminal development in the calf and that this improves absorptive capacity of the rumen, which is in accordance with our findings in the pre-weaning goat rumen [37, 38]. It has been reported that the intrinsic component of the INS in the GI of non-ruminants contains two ganglionated plexuses, the MP, located between the two muscle layers, and the submucosal plexus, located near the luminal side between the inner CM and the mucosa. The both two ganglionated plexuses contain a large quantity of NSs that arranged as concentric rings in the wall of the GI [34, 39, 40]. In the forestomach of ruminants, NSs were also found to accumulate in the both two ganglion [25–30]. However, our results showed that the NSs were present within the MP, while they were absent in the submucosal plexus. This is different from previous reports. Additionally, several studies focused on the number change of NSs per ganglion in the MP of the GI in ruminants or non-ruminants during postnatal development [25–30, 34–36, 41, 42]. Previous studies proposed that the amount of NSs per ganglion in the mouse myenteric and submucosal plexus ganglia decreases with age [43, 44]. In addtion, Pfannkuche et al. investigated that the number of NSs per ganglion of ovine rumen decreased significantly with age up to the ruminating stages where it remained stable [27]. Similarly, our study revealed that the density of NSs decreased with age in the MP of goats rumen before weaning. Furthermore, it has been proposed that the expression level of a neuroprotein could have strong physiological impacts. For instance, study showed that neuroprotein expression changes during intestinal inflammation in animals, leading to structural and functional alterations [36]. In particular, it was reported that antibiotic-induced microbiota dysbiosis affects the expression of nNOS [42]. In this study, the expression of the PGP9.5 protein was significantly reduced with age during rumen development and maturation. Therefore, it is speculated that a reduction in the number of NSs is a key reason for a decrease in the expression of PGP9.5-positive nerves. Interestingly, in rumen epithelium, the noticeable increase of MOD of PGP9.5-positive NFs indicated that some NFs, initially labeled in the rumen walls, or their progeny, may migrate towards the rumen epithelium. Epithelium of the rumen papillae, as the intestinal epithelial cell, define the largest exchange surface between the body and the external environment. Neunlist M et al. proposed a novel concept, the digestive ‘neuronal–glial–epithelial unit’: the nerves gave a central role to epithelial cells in the regulation of fluid and nutrient intake and this unit were also likely to regulated the passage of pathogens such as bacteria or immune cells [45]. This new concept may also apply to the rumen epithelium. The decreasing MOD of PGP9.5-positive NFs in the thickening ML may be related to the transformation of the different types of smooth muscle fibers with age [46, 47], although this requires further investigation.

In addition, INS development must be integrated with that of the various non-neural crest derived cells in guts that both influence INS signaling and show the stereotypical patterns of INS output [48–50]. Previously, it was suggested that functional coordination in the GI results from integrated communication between the ENS and “effector” cells such as ICs and smooth muscle cells. The tripartite communication has also been documented among enteric neurons, ICs and smooth muscle cells in the muscularis externa in the INS [51–53]. For example, the IC network, is actively involved in homeostatic mechanisms that maintain enteric neurotransmission [9, 17, 52]. Therefore, this suggests that the structural integrity of this network and its intimate association with nerves play an important role in the neural functional integrity, ensuring normal operation of the GI. Similarly, the network of ICs was also formed by the interconnection between the protuberances and somata of ICs. We found that the IC network and the neural network interweaved in the rumen wall and these two networks were gradually completed with age. Alternatively, some studies suggested that most IC somata and their projections were in close proximity with NFs, forming functional coupling with the cells of the outer CM [23, 24, 52, 53]. In our study, the somata and projections of several ICs closely surrounded each ganglionic soma and each myenteric nerve trunk. In addition, the IC projections gradually extended and lengthened, eventually wrapping tightly around the nerve trunks to form an almost homogenous and seamless ring-boundary layer. The space between the ring-boundary layer and the nerve trunk decreased with age, suggesting that their coordinated relationship was enhanced. Additionally, a blood vessel was noticed close to the ring-boundary. Structure and morphology of the ring-boundary resembled to that of the blood brain barrier (a diffusion barrier), which filtrates influx of compounds from blood to brain [54] and may maintain the stability of the microenvironment of NFs and NSs. Within the ML, the NFs were always parallel or in close proximity to the ICs and a close connection between NF, ICs and muscle cells was observed. This suggests that the ICs may take part in neurotransmission from NFs to smooth muscle by forming the close connections among them, which was investigated in the previous study [55]. This also indicates that the change of the neural distribution pattern during rumen development may be associated with ICs but the exact mechanism still needs to be explored.

In conclusion, the study of age-related neural rezoning showed that the goat rumen undergoes tremendous morphological changes during the pre-weaning period. Moreover, the relationships between NS/NF and IC were deciphered. We showed that the change of neural distribution pattern may lead to structural and functional coordination with ICs from birth to weaning, opening new perspectives in the understanding of INS postpartum development in ruminants.

## MATERIALS AND METHODS

### Animals

Twelve female Capra hircus ranging from 0 to 3 months (5-day-old, average weight=3.45 kg, n=4; 40-day-old, average weight=8.87 kg, n=4 and 70-day-old, average weight=22.63 kg, n=4) were used in this study. All female goats were obtained from a commercial farm and managed in a traditional control and treatment manner. Animals were deeply anesthetized using IV administration of sodium thiopental (10 mg/kg) and euthanized by an intracardiac injection of Tanax (0.5 ml/kg) [56]. The rumen samples were sampled immediately following euthanasia.

The rumen samples were collected immediately and placed in 10% neutral buffered formalin for fixation overnight and were then embedded in paraffin wax. 5 μm sections were then cut. The all goats was conducted according to the accepted international standards and was approved by the Ethics Committee for Animal Care and Use by the Science and Technology Agency of Jiangsu Province (SYXK (SU) 2010-0009). All efforts were done to minimize suffering of experimental animals.

### Light microscopy

The sections were stained with hematoxylin and eosin (Harry’s hematoxylin for 1.5 min and 1% eosin for 30 sec) and examined using an Olympus BX53 microscope.

### Immunofluorescence (IF)

For immunofluorescence, the paraffin sections underwent deparaffinization in an ethanol gradient followed by washing in PBS and then the slices were covered with 5% bovine serum albumin and sealed at 37°C for one hour. Then the sections were incubated with a pair of antibody: mouse anti-vimentin (1:100) (Boster BioTechnology, Wuhan, China) and rabbit anti-PGP9.5 (1:100) (Boster BioTechnology, Wuhan, China) or with just one antibody: rabbit anti-PGP9.5 (1:100) (Boster BioTechnology, Wuhan, China) overnight at 4°C. Following primary antibody application, all the samples were incubated with a secondary antibody for 1 h at 4°C and were rehydrated in PBS. The sections were incubated with DAPI and were stimulated under a fluorescent microscope over time. All the specimens were initially viewed with the use of a LED to visualize fluorescence under the microscope. Photos of each section of the specimen were taken at 40**–**400 magnification power. Finally, the Image-Pro Plus 6.0 software was used to calculate the density of positive nerves or ICs and GraphPad Prism5 was used to make the columns.

### Transmission electron microscopy (TEM)

The specimens were cut into small parts (1 mm^3^) prior to immersion in 2.5% glutaraldehyde in PBS (4°C, pH 7.4, 0.1 mol/L) during 24 h. Tissue was rinsed in PBS and post fixed during 60 min at room temperature using buffered 1% osmium tetroxide (Polysciences Inc. Warrington, PA). The samples were then dehydrated in ascending concentrations of ethyl alcohol, infiltrated with a propylene oxide–Araldite mixture and embedded in Araldite. The blocks were then sectioned using an ultramicrotome (ReichertJung, Wien, Austria), and the ultrathin sections (50 nm) were mounted on copper coated grids. The pieces were stained with 1% uranyl acetate and Reynold’s lead citrate for 20 min. Finally, specimens were examined and photographed using a high resolution diGI al camera (16 mega pixel) connected to the TEM, Hitachi H-7650 (Japan).

### Western blot

After tissue lysis, quantification of the protein supernatant was performed with the use of a BCA (bicinchoninic acid) protein assay kit. The samples were heated at 100°C for 10 min, and 8 μL of each extract were loaded on a 10% SDS-polyacrylamide gel. Proteins were transferred to a PVDF membrane (Millipore Bedford, MA), and the membrane was blocked for 2 h with 5% (w/v) skim milk in TRIS-buffer containing 0.5% of Tween 20 (TBS-T). The blots were then incubated with a polyclonal rabbit anti-PGP9.5 antibody (diluted 1:1000 in TBST, Boster, Wuhan, China) or anti-β-actin (diluted 1:10000, in TBST, Boster, Wuhan, China) overnight at 4°C. The membrane was then washed five times in TBST, followed by incubation with HRP-conjugated goat anti-rabbit IgG during 2 h. After another 5 washes in TBST, antigen-antibody complexes were visualized using an enhanced chemiluminescence detection kit. Each experiment was repeated three times. The size of the protein maker was 120 kDa (TaKaRa Dalian, China).
